# Supplementary material for: Mutations in PCYT2 disrupt etherlipid biosynthesis and cause a complex hereditary spastic paraplegia
Source: Brain. 2019 Oct 22;142(11):3382–97. doi: 10.1093/brain/awz291 (PMC6821184; doi:10.1093/brain/awz291)
Supplement: awz291_Supplementary_Data [file awz291_supplementary_data.zip › awz291-Suppl_data/Supplementary_Data4.zip!/Supplementary information 4 - plasma lipidomics.zip!/Plasma lipidomics/Control vs Patient1/Statistical plots and data/Cer[d] species/Volcano plot_alternative.pdf]

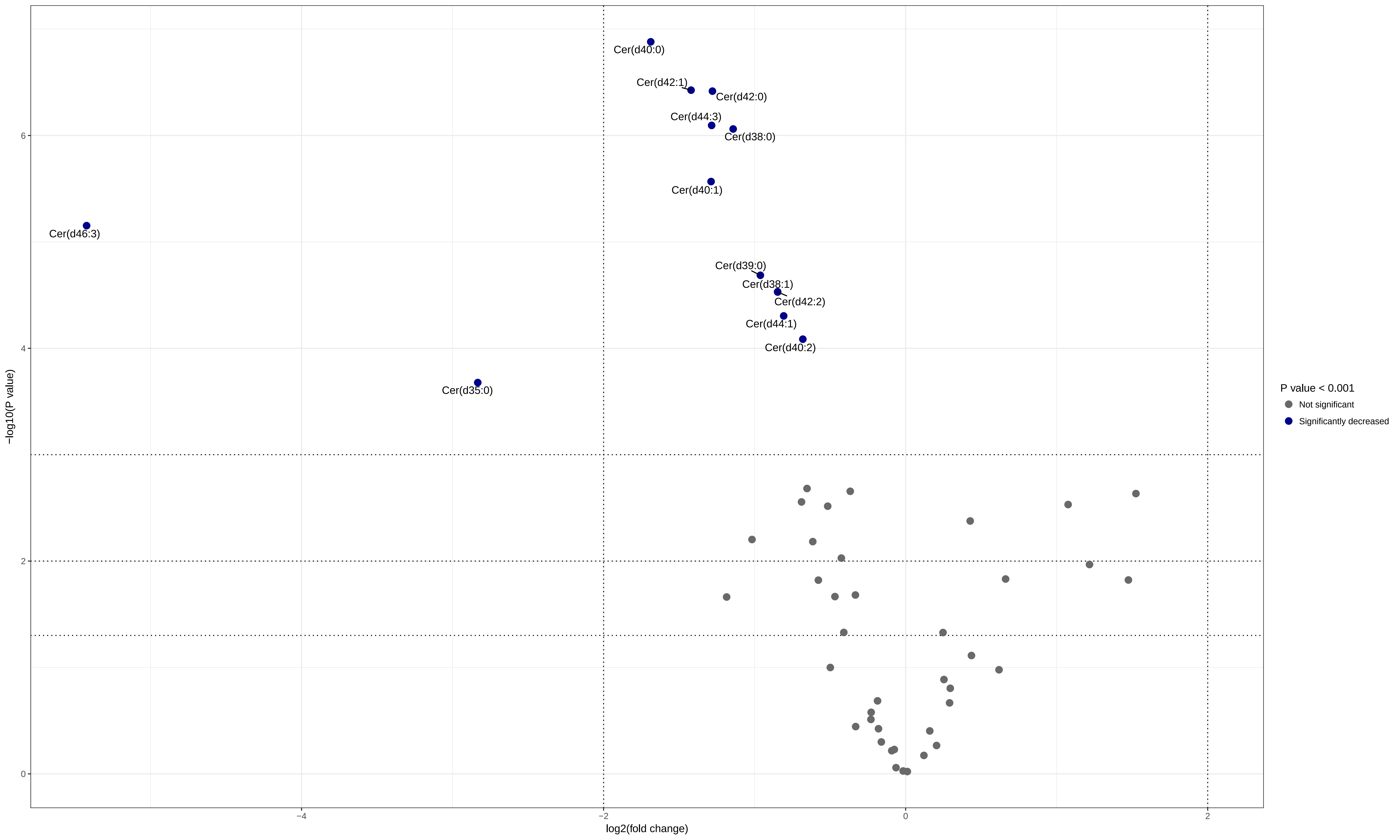
Cer(d40:0)
Cer(d42:1)
Cer(d42:0)
Cer(d44:3)
Cer(d38:0)
Cer(d40:1)
Cer(d46:3)
Cer(d39:0)
Cer(d38:1)
Cer(d42:2)
Cer(d44:1)
Cer(d40:2)
Cer(d35:0)
P value < 0.001
Not significant
Significantly decreased
−log10(P value)
log2(fold change)
6
4
2
0
−4
−2
0
2
